# Posture and mobility of the upper body quadrant and pulmonary function in COPD: an exploratory study^*^

**DOI:** 10.1590/bjpt-rbf.2014.0162

**Published:** 2016-04-08

**Authors:** Nuno Morais, Joana Cruz, Alda Marques

**Affiliations:** 1 School of Health Sciences (ESSLei), Polytechnic Institute of Leiria (IPL), Leiria, Portugal; 2 Respiratory Research and Rehabilitation Laboratory (Lab 3R), School of Health Sciences, University of Aveiro (ESSUA), Aveiro, Portugal

**Keywords:** body alignment, chronic obstructive pulmonary disease, musculoskeletal adaptations, physical therapy, respiratory function

## Abstract

**Background:**

There is limited evidence regarding interactions between pulmonary (dys)function, posture, and mobility of the upper body quadrant in patients with chronic obstructive pulmonary disease (COPD).

**Objectives:**

This exploratory study aimed to investigate whether postural alignment and mobility of the upper quadrant are related to changes in pulmonary function and compare such variables between patients with COPD and healthy individuals.

**Method:**

Fifteen patients with COPD (67.93±9.71yrs) and 15 healthy controls (66.80±7.47yrs) participated. Pulmonary function (FEV_1_, FVC) was assessed with spirometry. Alignment and mobility of the head, thoracic spine, and shoulder were assessed using digital photographs. Pectoralis minor muscle (PmM) length and thoracic excursion were assessed with a measuring tape. Groups were compared and linear regression analyses were used to assess potential relationships between postural and mobility variables and pulmonary function.

**Results:**

Patients with COPD were more likely to have a forward head position at maximal protraction (28.81±7.30º vs. 35.91±8.56º, p=0.02) and overall mobility of the head (21.81±10.42º vs. 13.40±7.84º, p=0.02) and a smaller range of shoulder flexion (136.71±11.91º vs. 149.08±11.58º, p=0.01) than controls. Patients’ non-dominant PmM length and maximal head protraction were predictors of FEV_1_ (r^2^_adjusted_=0.34). These variables, together with the upper thoracic spine at maximal flexion and thoracic kyphosis at maximal extension, were predictors of FVC (r^2^_adjusted_=0.68).

**Conclusion:**

Our findings suggest that impaired pulmonary function is associated with muscle length and mobility adaptations. Further studies are needed to understand the underlying mechanisms and clinical value of these relationships.

## Bullet points

- Differences in head and shoulder mobility were found between groups.
- Pulmonary function was associated with muscle length and mobility adaptations.
- Further research is needed to support findings.

## Introduction

Chronic Obstructive Pulmonary Disease (COPD) is a progressive respiratory disease that affects 210 million people worldwide^1^. It is caused by a mixture of small airways disease (obstructive bronchiolitis) and parenchymal destruction (emphysema), which reduce the elastic recoil of the lung and increase airways resistance^2^. The main characteristic of COPD is persistent expiratory airflow that results in air trapping and, consequently, hyperinflation^3^. Hyperinflation develops early in the course of the disease and has been considered the key mechanism for exertional dyspnea^2^. Previous studies have shown that secondary postural changes of the chest wall and impaired chest mobility wall may occur in response to lung hyperinflation and increased work of breathing, further limiting the mechanical effectiveness of the rib cage inspiratory muscles^4^ ^-^ ^6^. Thus, improving postural alignment and mobility of the chest wall, spine, and shoulders is now part of the recommendations for comprehensive pulmonary rehabilitation programs^7^. However, the possible effect of such interventions in patients with COPD is uncertain because little is known about the interaction between pulmonary (dys)function, posture, and mobility of the upper body quadrant (i.e. head, cervical and thoracic spines, thorax, and upper limb). A recent systematic review, aimed at evaluating the effect of interventions targeted at the musculoskeletal structures on the pulmonary function of patients with COPD^8^, found only 7 published studies on this subject, some of them with encouraging findings. For example, one study assessed 10 patients and found that applying a hold-relax technique to the pectoralis major muscle improved both vital capacity and shoulder range of motion (ROM) by approximately 10%^9^. Other studies showed that applying manipulative therapy to the upper quadrant significantly improved total lung capacity^10^ and forced expiratory volume in 1 second (FEV_1_)^11^. Despite these interesting results, evidence of musculoskeletal interventions as an adjunctive management approach for COPD is lacking and more exploratory research is needed to better understand the nature and extent of musculoskeletal changes in patients with COPD and their potential relationship with pulmonary function^8^.

Therefore, the aim of this study was to explore whether postural alignment and mobility variables of the upper quadrant are associated with changes in pulmonary function and compare such variables between patients with COPD and healthy individuals.

## Method

### Design

A cross-sectional exploratory study was conducted after receiving approval from the Ethics Committee of the Central Regional Health Administration, Coimbra, Portugal (2011-02-28). The study was reported following the Strengthening the Reporting of Observational Studies in Epidemiology (STROBE) guidelines^12^.

### Participants

Physicians of one primary care center identified adults with a clinical diagnosis of COPD, according to the Global Initiative for Chronic Obstructive Lung Disease (GOLD) criteria^2^. Patients were included if they were: ≥18 years old; clinically stable over the past month; living in the community; able to walk independently without an assistive device; and able to follow instructions. Patients were excluded if they had: thoracic or abdominal surgery in the previous year; recent/recurrent musculoskeletal injury to the upper body quadrant; previous mastectomy; other severe musculoskeletal, systemic, neurological, or cardiovascular disorders that could interfere with the measurements; or cognitive impairment.

Physicians informed patients about the study and asked about their willingness to participate. Patients who agreed to participate were contacted by researchers to schedule an appointment in the primary care center, from March to July 2013. All participants reported to have taken their usual prescribed medications before participation.

The control group included healthy adults who volunteered to participate in the study. Eligibility criteria were the same as the COPD group, with the addition of normal pulmonary function values and absence of respiratory complaints. There was an attempt to recruit healthy adults within the same age range of patients (51-81 years), since postural variables of the upper body quadrant may change with age^13^; nevertheless, formal age-matching was not performed. Before data collection, more information about the study was provided and written informed consent was obtained.

### Outcome measures

Participants were asked about socio-demographic information and current respiratory medication. Anthropometric and pulmonary function data were then collected, followed by the measurement of variables related to postural alignment and mobility of the upper quadrant. These were assessed in a random sequence.

### Anthropometrics

Body mass and height were read and recorded to the nearest 0.5 Kg and 0.5 cm, respectively, with participants barefooted. Body mass index (BMI) was calculated.

### Pulmonary function

Pulmonary function (FEV_1_ and forced vital capacity–FVC) was assessed using a portable spirometer (MicroLab3500, CareFusion, Kent, UK)^14^. COPD grades were determined to characterize the sample, according to the Global Initiative for Chronic Obstructive Lung Disease (GOLD) criteria^2^: mild (FEV_1_≥80% predicted), moderate (50%≤FEV_1_<80% predicted), and severe-to-very-severe (FEV_1_<50% predicted) COPD.

### Alignment and mobility of the upper quadrant

#### Head, thoracic spine, and shoulder

Alignment and mobility of the head, thoracic spine and shoulders were assessed using lateral digital photographs. A 12-megapixel camera (PowerShot SX200, Canon, Tokyo, Japan) was set on a tripod with a multi-angle bubble level and positioned at 1.50 m from a cross mark drawn on the ground. Participants were aligned perpendicular to the camera and instructed to stand in the upright position as described elsewhere^15^. One researcher attached 5 rigid plastic screw anchors (height, 40 mm; base, 10 mm) over the skin of the spinous processes of the 7^th^ cervical^13^ and the 1^st^, 4^th^, 8^th^ and 12^th^ thoracic^16^ vertebrae using double-sided tape (Figures 1S and 2S of the supplementary material). These markers, representing body landmarks, were used to determine angular measurements of the head and (upper and lower) thoracic spine. Participants received specific and standardized instructions about the testing positions: neutral and maximal positions of head protraction and retraction, thoracic flexion and extension, and shoulder flexion. They followed a standardized protocol to obtain a natural and self-balanced upright posture^15^ and were photographed from the right side in each testing position. To obtain maximal head protraction and retraction, participants were asked to move their head as far as they could forward or backwards while fixing their gaze on a point straight ahead on the wall and maintaining an upright body posture. Similar instructions were provided to obtain maximal thoracic flexion and extension and shoulder flexion based on previous research^17^ ^,^ ^18^. Participants returned to the neutral position between maximal trials. A practice trial was performed with the supervision of one researcher for participants to be familiar with the testing procedures (instructions, movements, and photographic image capture) before data collection. The same researcher supervised all data recording to ensure that tasks were following standardized procedures.

#### Pectoralis minor muscle (PmM) length

The PmM is a key muscle in postural misalignment and impaired mobility of the upper quadrant^19^ ^,^ ^20^. Its shortening has been associated with reduced pulmonary function^21^ and found in patients with chronic respiratory disease^22^. Assessment was performed with a measuring tape and comprised the distance between the coracoid process and the inferior border of the 4^th^ rib, in the standing position with arms alongside the trunk^20^. This methodology has shown good agreement between tape, caliper, and electromagnetic motion tracking device measurements (ICC 0.82-0.87, standard error of ~0.3 cm), an excellent intrarater reliability (ICC=0.98-0.99), and a standard error of the measurement (SEM) ranging from 0.29 to 0.32 cm^23^. Both sides were assessed.

#### Thoracic excursion

Chest mobility was assessed by thoracic excursion, i.e. the difference between thoracic circumference at peak inspiration and expiration in the standing position with arms alongside the trunk^24^. The reliability of this methodology for upper and lower thoracic excursions is high (ICC=0.84-0.91)^24^. One researcher measured the thoracic circumference with a measuring tape held around the chest on two levels: upper and lower thorax. Upper thoracic excursion was assessed by placing the tape on the 3^rd^ intercostal space at the midclavicular line and the 5^th^ thoracic spinous process. Lower thoracic excursion was measured at the tip of the xiphoid process and the 10^th^ thoracic spinous process. Participants were asked to hold their breath at peak inspiration and expiration for data collection^24^. A practice trial was allowed for participants to be familiar with testing procedures.

### Data reduction

#### Photographs

Photographs were analyzed using a computer-assisted digitizing software (Osirix Imaging v5.0.2, Pixmeo SARL, Switzerland).

Head alignment and mobility were measured as the acute angle between a line joining the C7 marker to the tragus of the ear and a horizontal line (Figure 1S of the supplementary material)^13^. It determines the amount of forward head positioning; the smaller the angle, the more forward is the head. This measure has demonstrated reasonable reproducibility and agreement, with ICC values ranging from 0.39 to 0.87^13^ ^,^ ^25^ ^,^ ^26^ and minimal detectable change (MDC) ranging from 5.13º to 9.81º^25^. Total mobility of the head was calculated as the difference between maximal protraction and retraction positions, in absolute values.

Alignment and mobility of the upper and lower thoracic spines were measured using the angles formed by the line passing the extremity of T1 and T4 markers and a vertical line and the line passing the extremity of T8 and T12 markers and a vertical line, respectively (Figures 1S and 2S of the supplementary material)^16^. Thoracic kyphosis was the sum of the upper and lower thoracic spine angles. This measuring system showed significant correlations with radiographic measurements for both thoracic kyphosis in the neutral position (r=0.76, P<.001) and maximal thoracic extension (r=0.69, P<.01). The coefficient of variation and SEM of photographic measurements have been found to be 4.8% and 0.5° for the neutral position and 7.8% and 0.6° for maximal thoracic extension^16^. Total mobility of the upper, lower, and total thoracic spine (i.e. thoracic kyphosis) were the difference between maximal flexion and extension, in absolute values.

Maximal shoulder flexion angle was determined using the standard goniometry landmarks, lateral epicondyle of the humerus, lateral midline of the thorax^27^, and the centroid of the virtual glenohumeral joint region (Figure 2S ^**^ of the supplementary material). A pilot study was previously conducted with 10 subjects (4 males, 70.5±10.3yrs) to assess reliability and agreement properties of this methodology. Excellent intrarater (ICC_2,1_=0.99, 95% confidence intervals [95%CI]=0.98-0.99, SEM=0.85º, MDC_95_=2.36º) and interrater (ICC_2,1_=0.98 95%CI=0.93-0.99, SEM=1.54º, MDC_95_=4.28º) reliability and agreement results were found.

### Statistical analysis

The Shapiro-Wilk and the Levene tests revealed that all quantitative data followed a normal distribution and that homoscedasticity could be assumed, respectively. Socio-demographic and clinical variables of the groups were compared using independent *t*-tests for normally distributed data and Chi-square tests for categorical data. Effect sizes (*d*) quantified the strength of between-group differences. A *d*≥0.2 indicated a small, 0.2>*d*≥0.5 a medium, and 0.5>*d*≥0.8 a large effect^28^. Multiple linear regression models (stepwise and forward methods) were used to assess relationships between FEV_1_ and FVC absolute values (dependent variables) and alignment and mobility of the upper quadrant (independent variables), in each group. Only significant predictors were retained in the models. The r^2^ was calculated to indicate how well the data fitted in the model, and the r^2^_adjusted_ using Stein’s formula^29^ was computed to estimate how well the derived equation would predict on other samples from the same population. Model stability was assessed by a bootstrap method based on 1000 replicates of the initial dataset. Assumptions of multiple linear regression models were tested by checking the normal distribution (Shapiro-Wilk test and the analysis of the P–P and the Q–Q plots), the homoscedasticity (analysis of the residuals plot), and independence (Durbin–Watson test) of the residuals, plus multicollinearity of the predictors (Pearson’s correlation, tolerance, and variance inflation factor). Analyses were conducted using SPSS v20.0 (IBM, Armonk, NY, USA) and G*Power v3.1.3 (G*Power, Kiel, Germany). The level of significance was set at 0.05. There were no missing data in the database and assumptions for an appropriate application of multiple linear regressions were assumed.

## Results

### Participants

Twenty-five patients with COPD were invited to participate; however, 2 did not meet the eligibility criteria (reasons: inability to walk without an assistive device, presence of thoracolumbar scoliosis), 3 refused to participate and 5 failed to attend the appointment. Hence, 15 patients (13 males) completed the study (Figure 3S of the supplementary material). Sixteen healthy individuals volunteered to participate in the study. One presented abnormal spirometry values and was not included.

Baseline characteristics of the groups are described in Table 1. Significant differences between groups were found for the mean FEV_1_ (absolute value=0.55L, 95%CI=0.16L-0.95L; percentage predicted=34.67% 95%CI=22.40%-46.93%) and FVC (percentage predicted=16.20% 95%CI=3.99%-28.41%).

**Table 1:** Socio-demographic and clinical characteristics of patients with COPD and healthy controls.

	**Patients with COPD (n=15)**	**Healthy controls (n=15)**	**p-value**
Age (*years*)	67.93±9.71	66.80±7.47	0.72
Gender (*male*), n (%)	13 (86.7%)	7 (46.7%)	0.05
Height (*cm*)	166.00±6.18	160.87±8.58	0.07
Weight (*Kg*)	77.37±17.95	72.47±10.39	0.37
BMI (*Kg/m^2^*)	28.00±5.87	28.01±3.30	0.99
FEV_1_			
Value (*L*)	1.72±0.52	2.27±0.53	0.01^*^
% pred	66.00±17.77	100.67±14.90	<0.01^*^
FVC			
Value (*L*)	2.57±0.77	2.58±0.62	0.97
% pred	75.33±17.78	91.53±14.74	0.01^*^
GOLD grade, n (%)			
Mild COPD	2 (13.3%)	-	
Moderate COPD	11 (73.4%)	-	
Severe-to-very-severe COPD	2 (13.3%)	-	
Respiratory medication, n(%)			
Long-acting beta2-agonists	1 (6.67%)	-	
Short-acting beta2-agonists	3 (20.00%)	-	
Long-acting anticholinergics	4 (26.67%)	-	
Inhaled corticosteroids	1 (6.67%)	-	
Combination of long-acting beta2-agonist plus corticosteroids	4 (26.67%)	-	

Values are shown as mean±SD, unless otherwise indicated. BMI: body mass index; FEV_1_: forced expired volume in 1 second; FVC: forced vital capacity; GOLD: Global Initiative for Chronic Obstructive Lung Disease; % pred: percentage predicted.

* Significant at p<0.05.

### Alignment and mobility of the upper quadrant

Tables 2 and 3 present the alignment and mobility of the head, thoracic spine, and shoulders, PmM length and thoracic excursion of both groups. Compared to controls, patients with COPD presented, on average, a more forward head at maximal protraction (7.09º 95%CI=1.15º-13.04º, p=0.02, *d*=0.89), a higher overall mobility of the head (8.42º 95%CI=1.52º-15.31º, p=0.02, *d*=0.91), and a smaller range of shoulder flexion (12.37º 95%CI=3.58º-21.16º, p=0.01, *d*=1.05). No significant between-group differences were found for the thoracic circumferences and excursion.

**Table 2:** Alignment and mobility of the head, thoracic spine and shoulder, and pectoralis minor muscle length of patients with COPD and healthy controls.

	**Position/side**	**Patients with COPD (n=15)**	**Healthy controls (n=15)**	**Mean difference** **[95% CI]**	**p-value**	**Effect size *d***	**Observed power**
*Alignment and mobility* (°)							
Head	Neutral position	45.54±9.16	43.16±4.72	2.38 [–3.07 to 7.83]	0.38	0.33	0.14
	Maximal protraction	28.81±7.30	35.91±8.56	–7.09 [–13.0 to -1.15]	0.02^*^	0.89	0.65
	Maximal retraction	50.62±8.62	47.17±9.83	3.46 [–3.46 to 10.37]	0.31	0.37	0.16
	Total mobility	21.81±10.42	13.40±7.84	8.42 [1.52 to 15.31]	0.02^*^	0.91	0.67
Upper thoracic spine	Neutral position	21.68±6.88	22.58±4.58	–0.90 [–5.27 to 3.47]	0.68	0.15	0.07
	Maximal flexion	36.49±8.62	40.42±8.17	–3.93 [–10.22 to 2.35]	0.21	0.47	0.24
	Maximal extension	13.13±7.05	17.01±7.23	–3.87 [–9.21 to 1.47]	0.15	0.54	0.30
	Total mobility	23.35±10.28	23.41±10.64	–0.06 [–7.88 to 7.77]	0.99	0.01	0.05
Lower thoracic spine	Neutral position	–10.73±6.78	–8.82±4.13	-1.91 [-6.11 to 2.29]	0.36	0.34	0.15
	Maximal flexion	–2.00±9.64	0.95±7.31	-2.95 [-9.35 to 3.46]	0.35	0.34	0.15
	Maximal extension	-14.82±7.28	–11.61±4.31	–3.22 [–7.69 to 1.26]	0.15	0.54	0.30
	Total mobility	12.83±7.86	12.55±7.47	0.27 [–5.46 to 6.01]	0.92	0.04	0.05
Thoracic kyphosis	Neutral position	33.08±8.65	31.40±6.90	1.67 [–4.18 to 7.52]	0.56	0.21	0.09
	Maximal flexion	44.01±11.50	46.50±9.47	–2.49 [–10.37 to 5.38]	0.52	0.24	0.10
	Maximal extension	28.00±9.05	28.61±9.67	–0.61 [–7.62 to 6.39]	0.86	0.07	0.05
	Total mobility	16.01±14.22	17.99±12.31	–1.98 [–11.93 to 7.96]	0.69	0.15	0.07
Shoulder joint	Maximal flexion	136.71±11.91	149.08±11.58	–12.37 [–21.16 to –3.58]	0.01^*^	1.05	0.79
*PmM length* (*cm*)	Dominant	16.51±1.50	15.91±1.36	0.61 [–0.47 to 1.68]	0.26	0.42	0.20
	Non-dominant	16.77±1.75	15.65±1.44	1.13 [–0.07 to 2.32]	0.06	0.70	0.46

Values are shown as mean±SD, unless otherwise indicated. PmM: Pectoralis minor muscle; 95% CI: 95% confidence intervals.

* Significant at p<0.05.

**Table 3:** Thoracic circumference at peak inspiration and expiration and thoracic excursion, measured at the upper and lower thorax, in patients with COPD and healthy controls.

	**Patients with COPD (n=15)**	**Healthy controls (n=15)**	**Mean difference** **[95% CI]**	**p-value**	**Effect size *d***	**Observed power**
**Upper thorax (cm)**						
Thoracic circumference at PI	104.32±10.62	100.94±6.15	3.38 [–3.11 to 9.87]	0.30	0.39	0.18
Thoracic circumference at PE	101.97±11.24	98.33±5.72	3.64 [–3.03 to 0.31]	0.27	0.41	0.19
Thoracic excursion	2.35±1.38	2.61±1.06	–0.26 [–1.18 to 0.66]	0.57	0.21	0.09
**Lower thorax (cm)**						
Thoracic circumference at PI	101.46±11.17	97.94±5.25	3.52 [–3.13 to 10.16]	0.28	0.40	0.18
Thoracic circumference at PE	98.59±11.72	94.32±4.82	4.27 [–2.59 to 11.13]	0.21	0.48	0.25
Thoracic excursion	2.86±1.24	3.62±1.	–0.75 [–1.85 to 0.35]	0.17	0.52	0.28

Values are shown as mean±SD, unless otherwise indicated. PI: peak inspiration; PE: peak expiration; 95% CI: 95% confidence intervals.

### Relationships between pulmonary function and upper quadrant variables

#### Predictors of FEV_1_

Non-dominant PmM length and maximal head protraction predicted patients’ FEV_1_ (p=0.01), explaining 34% of its variance (r^2^_adjusted_=0.34, Table 4). In controls, FEV_1_ was predicted by thoracic circumference of the lower thorax at peak inspiration, head posture in neutral position, and upper thoracic spine at maximal extension (p<0.01, r^2^_adjusted_=0.68).

**Table 4:** Predictors of FEV_1_ (Liters) in patients with COPD and healthy controls, using multiple regression analysis.

	**B [95% CI]**	**β**	**p-value**
**Patients with COPD (n=15)**			
Non-dominant PmM length	0.193 [0.065 to 0.320]	0.652	0.01
Maximal head protraction	0.038 [0.007 to 0.068]	0.534	0.01
**Healthy controls (n=15)**			
Thoracic circumference of the lower thorax at PI	0.070 [0.041 to 0.099]	0.688	<0.01
Head posture in neutral position	0.100 [0.058 to 0.143]	0.890	<0.01
Upper thoracic spine at maximal extension	–0.041 [–0.069 to –0.014]	–0.562	0.01

Patients with COPD: Constant=-2.602. F(2,12)=7.464; p=0.01; r^2^=0.55; r^2^_adjusted_=0.34. Healthy controls: Constant=-8.170; F(3,11)=16.660; p<0.01; r^2^=0.82; r^2^_adjusted_=0.68. PI: peak inspiration; PmM: Pectoralis minor muscle; FEV_1_^:^ Forced Expired Volume in the First second.

#### Predictors of FVC

In patients with COPD, non-dominant PmM length, upper thoracic spine alignment at maximal flexion, maximal head protraction, and thoracic kyphosis at maximal extension were predictors of FVC (p<0.01), explaining 72% of its variance (r^2^_adjusted_=0.72, Table 5). In controls, predictors of FVC were similar to those of FEV_1_ (p<0.01, r^2^_adjusted_=0.68).

**Table 5:** Predictors of FVC (Liters) in patients with COPD and healthy controls, using a multiple regression analysis.

	**B [95% CI]**	**β**	**p-value**
**Patients with COPD (n=15)**			
Non-dominant PmM length	0.423 [0.287 to 0.560]	0.958	<0.01
Upper thoracic spine at maximal flexion	0.045 [0.022 to 0.069]	0.505	<0.01
Maximal head protraction	0.047 [0.018 to 0.076]	0.441	0.01
Thoracic kyphosis at maximal extension	0.033 [0.007 to 0.058]	0.382	0.02
**Healthy controls (n=15)**			
Thoracic circumference of the lower thorax at PI	0.090 [0.056 to 0.123]	0.761	<0.01
Head posture in neutral position	0.101 [0.051 to 0.151]	0.770	0.01
Upper thoracic spine at maximal extension	–0.043 [–0.075 to –0.010]	–0.498	0.01

Patients with COPD: Constant=–8.442. F(4,10)=17.044; p<0.01; r^2^=0.87, r^2^_adjusted_=0.72. Healthy controls: Constant=–9.835. F(3,11)=16.633; p<0.01; r^2^=0.82; r^2^_adjusted_=0.68. PI: peak inspiration; PmM: Pectoralis minor muscle; FVC: Forced Vital Capacity.

## Discussion

This was the first study exploring the relationships between pulmonary function and alignment and mobility of the upper quadrant in patients with COPD and healthy controls. Patients with COPD presented significant differences in head and shoulder mobility compared to controls. Moreover, predictors of pulmonary function differed between groups. In patients, non-dominant PmM length, head protraction, and thoracic spine mobility were strongly related to pulmonary function. In controls, pulmonary function was mostly associated with head posture at rest and spinal and chest mobility. Although exploratory, these findings suggest that patients with COPD may show adaptations in mobility of the upper quadrant related to impaired pulmonary function, which may be important to consider when developing rehabilitation interventions.

No significant between-group differences were found in the alignment of the upper quadrant in the neutral position. While the assessment of posture and mobility of the upper quadrant has been overlooked in COPD, there is a general belief that individuals with chronic respiratory diseases present postural changes, specifically forward head posture and thoracic kyphosis^30^. The present findings do not seem to support this assumption, suggesting that COPD may not significantly affect patients’ postural alignment at rest. Similar results were found in the study of Dias et al.^6^, using comparable body landmarks to assess the angular position of the head and thoracic kyphosis but a different equipment to capture and determine upper body posture and motion, and a sample with a higher percentage of patients with severe COPD. Nevertheless, patients presented a significantly higher ROM in head protraction and overall head mobility when compared to healthy individuals. The mechanisms underlying these changes are unknown; however, they are likely to be morphofunctional adaptations to the disease, since head protraction was one of the variables associated with pulmonary function in patients (but not in controls). Further research is needed to understand the role of head mobility in COPD adaptive mechanisms.

Patients with COPD showed less shoulder flexion ROM than controls. Previous studies found that arm elevation, in these patients, results in changes in the breathing pattern and increased metabolic demands. These changes are assumed to be related to reduced efficiency of respiratory mechanics and the dual activity of some rib cage muscles, which have to sustain the arm at an elevated position and act as accessory respiratory muscles simultaneously^31^ ^-^ ^35^. The increased ventilatory and metabolic demands have also been found in patients with COPD while performing daily living activities requiring arm elevation^36^. Thus, a reduction in shoulder flexion, as found in this study, may have important clinical implications for daily functioning. To overcome this limitation, upper limb training is often recommended as part of an exercise regimen within pulmonary rehabilitation^7^. However, the optimal training approach remains undetermined and it is not yet clear whether specific gains in upper limb function translate into improvements in broader outcomes such as quality of life^7^. It is important to note that, in this study, shoulder flexion was a voluntary/active movement. Therefore, it was not possible to isolate the contribution of passive (ligaments, capsule) and active (muscles) components to the movement. For this reason, it is unknown whether shoulder flexion ROM is reduced *per se* in patients with COPD or if only active movement is limited.

In patients with COPD, PmM length on the non-dominant side and maximal head protraction were positively associated with FEV_1_. This indicates that, with pulmonary function decline, PmM length and head protraction ROM are reduced. Moreover, these variables, along with thoracic spine mobility, were predictors of FVC. These results suggest that interventions targeted at improving head and thoracic mobility and muscle length of the upper quadrant, such as muscle and joint flexibility exercises, may have the potential to improve patients’ pulmonary function. Previous studies evaluating the effects of techniques in joint mobility and muscle length of the upper quadrant support this practice^9^ ^-^ ^11^. Hence, improving joint and muscle flexibility of patients with COPD should not only be regarded as part of a comprehensive exercise regimen^7^, but also be considered as a possible therapy to improve pulmonary function. Further research is needed for a better understanding of the underlying mechanisms and clinical value of the relationships between mobility and muscle length changes of the upper quadrant and pulmonary function.

Non-dominant PmM length provided the largest contribution to the regression equations of pulmonary function. Although PmM has been acknowledged as one of the inspiratory accessory muscles^37^, its importance in COPD has not been studied. It is believed that the relationship between changes in muscle length and pulmonary function may be attributable to the mechanical disadvantage of rib cage inspiratory muscles (including the PmM) due to hyperinflation. Hyperinflation places these muscles in a shortened position, making the rib cage stiffer and difficult to expand^38^. The fact that only the non-dominant PmM was a predictor of pulmonary function is not clearly understood. Stronger correlations were found between pulmonary function and the non-dominant PmM when compared with the dominant side (0.53-0.60 and 0.43-0.49, respectively – data not shown in the Results), which may explain in part the findings. Additionally, it is possible that PmM length adaptations of the dominant side are more related to chronic exposure to upper limb activities of daily living^39^ than to pulmonary function. Further research is needed to clarify this issue/topic.

## Study limitations

This study had some limitations that should be acknowledged. Given its exploratory nature, sample sizes were relatively small, and most patients were in the moderate COPD grade. The generalizability of findings is then limited to patients with similar characteristics. Preliminary data reported here may serve as a base for sample size determination in larger scale studies. The non-dominant PmM was one predictor of patients’ pulmonary function; nevertheless, other respiratory muscles involved in both ventilatory and non-ventilatory activities (e.g. sternocleidomastoid, scalene) may also influence pulmonary function. This should be addressed in future studies. Gender differences between groups were in the borderline of statistical significance, which could have influenced the results. However, previous studies have suggested that gender is not a factor of variation in posture and kinematics of the upper quadrant^13^ ^,^ ^40^. In this study, no significant gender differences were found for posture and mobility (data not shown). Another limitation of this study is that measures of mobility of head, thoracic spine, rib cage, and shoulders were based on calculations from the positioning of markers placed over the skin. The accuracy and precision of these measures might have been affected from errors originated in marker positioning and motion of the skin (and markers) with respect to the underlying bone during movement of the segments. Nonetheless, these errors are often of relatively low magnitude and importance in most clinical situations^18^ ^,^ ^41^ ^,^ ^42^. Finally, this was a cross-sectional study, thus findings cannot demonstrate an actual cause-effect relationship. Longitudinal studies are needed.

In summary, patients with COPD presented impaired pulmonary function associated with pectoralis minor muscle length and mobility of the upper quadrant (i.e. head protraction and thoracic spine), possibly as musculoskeletal adaptations to the chronic respiratory condition. Nevertheless, this was a small exploratory study and further and more definitive research is needed to support the findings.

## Supplementary Material

Figure 1S. Angle measurements of the head (α), upper (β) and lower (γ) thoracic spines in the neutral position

Figure 2S. Angle measurement of the shoulder flexion (ω) at maximal arm elevation

Figure 3S. Participants’ flow diagram
