# Supplementary figures and images for: Posture and mobility of the upper body quadrant and pulmonary function in COPD: an exploratory study
Source: Braz J Phys Ther. 2016 Apr 8;20(4):345–54. doi: 10.1590/bjpt-rbf.2014.0162 (PMC5015673; doi:10.1590/bjpt-rbf.2014.0162)

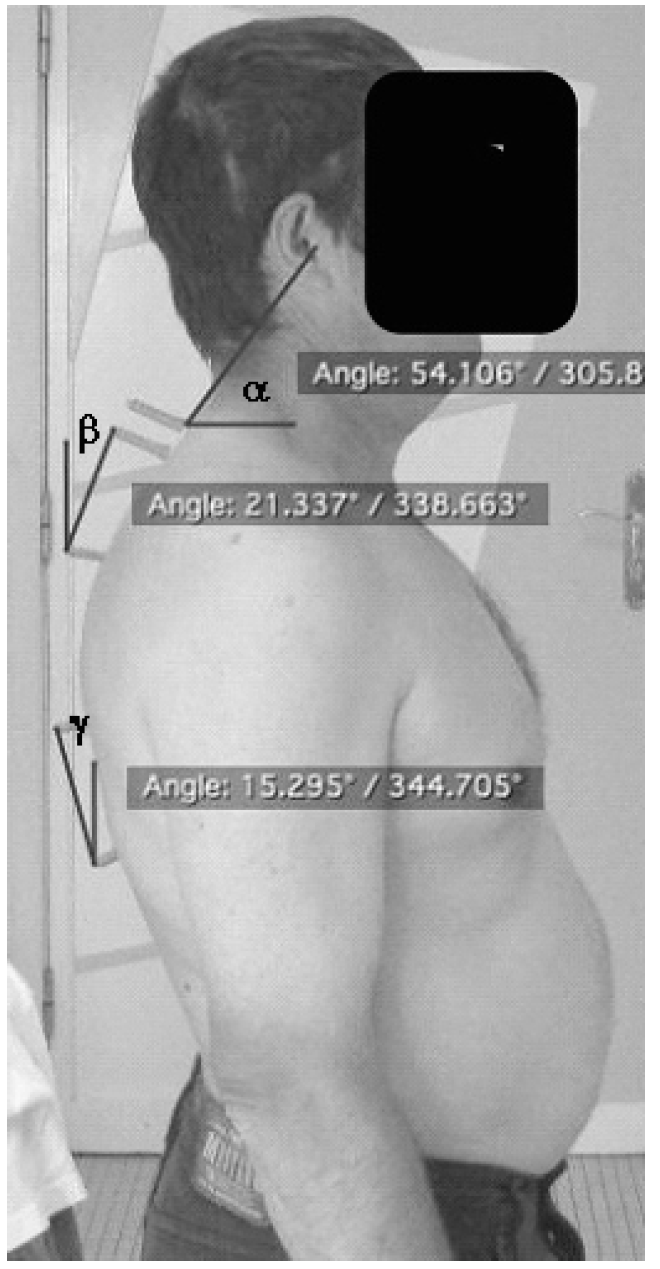

Supplement: Figure 1S. [file 1413-3555-rbfis-bjpt-rbf20140162-s1.tif]

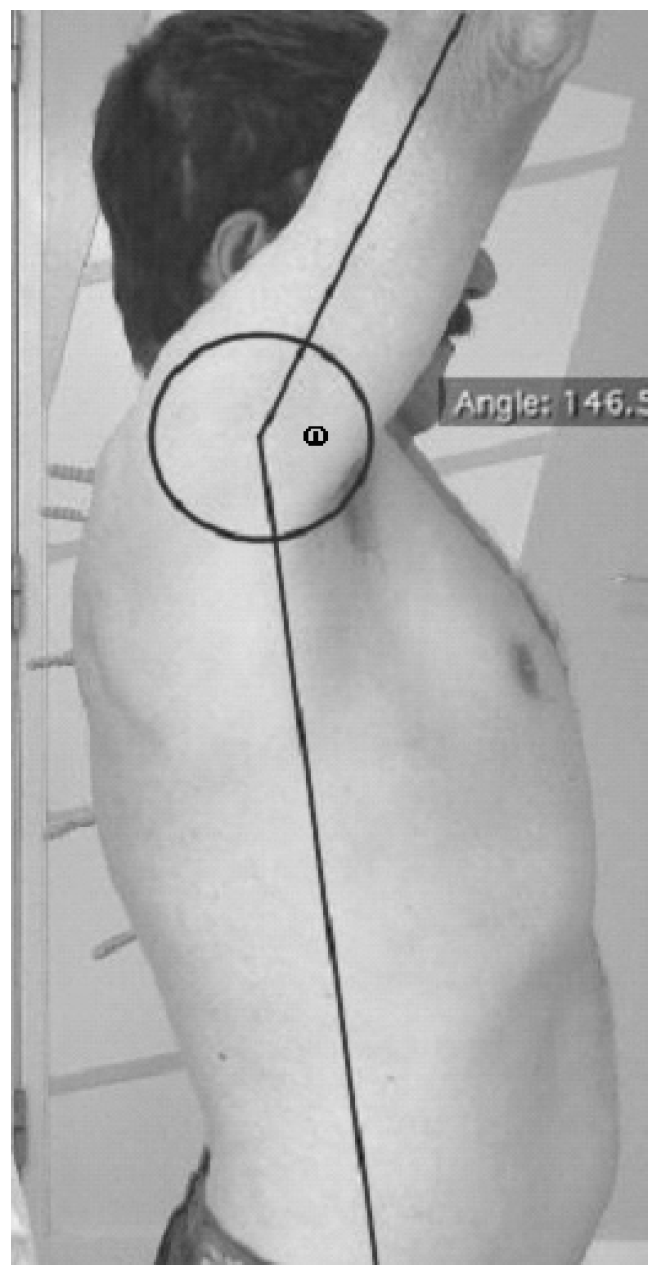

Supplement: Figure 2S. [file 1413-3555-rbfis-bjpt-rbf20140162-s2.tif]

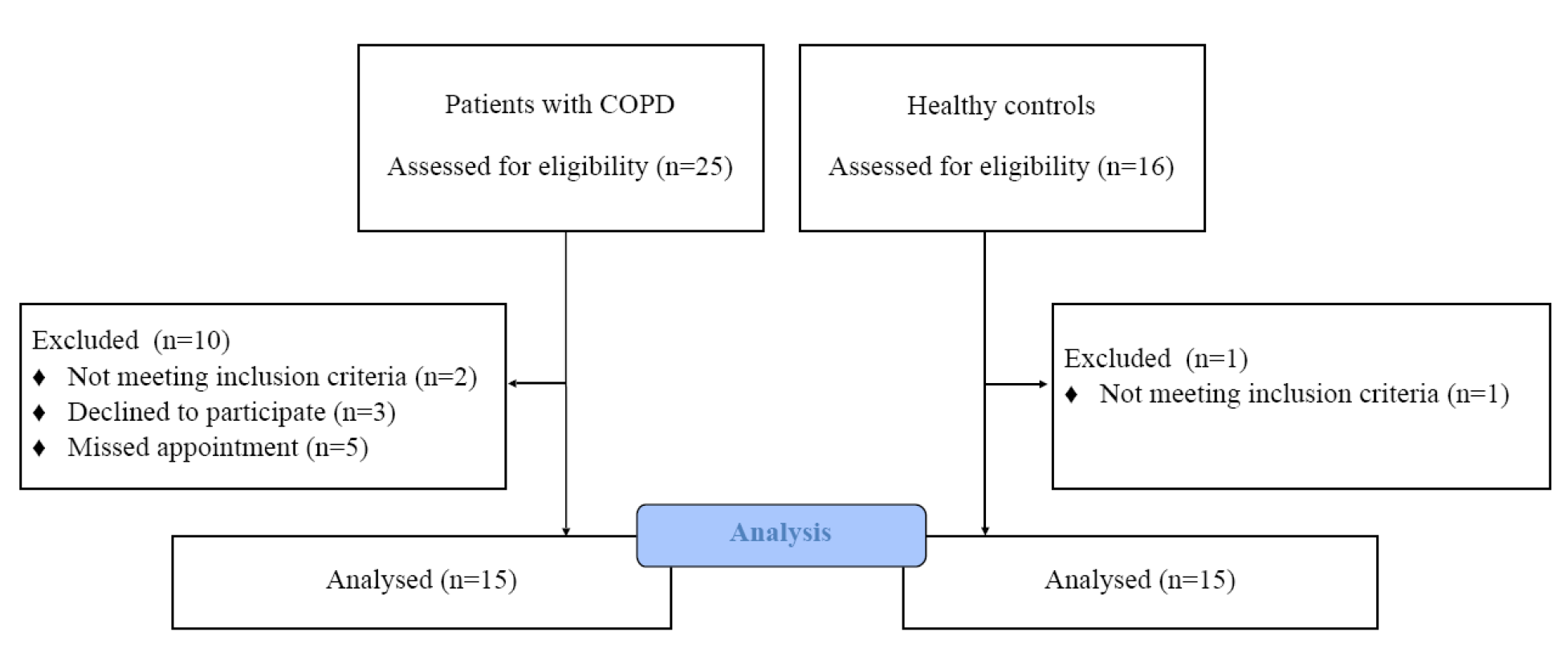

Supplement: Figure 3S. [file 1413-3555-rbfis-bjpt-rbf20140162-s3.tif]
